# Ensemble of Template-Free and Template-Based Classifiers for Protein Secondary Structure Prediction

**DOI:** 10.3390/ijms222111449

**Published:** 2021-10-23

**Authors:** Gabriel Bianchin de Oliveira, Helio Pedrini, Zanoni Dias

**Affiliations:** Institute of Computing, University of Campinas, Campinas 13083-852, Brazil; helio@ic.unicamp.br (H.P.); zanoni@ic.unicamp.br (Z.D.)

**Keywords:** protein secondary structure prediction, deep learning, machine learning, BLAST, ensemble

## Abstract

Protein secondary structures are important in many biological processes and applications. Due to advances in sequencing methods, there are many proteins sequenced, but fewer proteins with secondary structures defined by laboratory methods. With the development of computer technology, computational methods have (started to) become the most important methodologies for predicting secondary structures. We evaluated two different approaches to this problem—driven by the recent results obtained by computational methods in this task—(i) template-free classifiers, based on machine learning techniques; and (ii) template-based classifiers, based on searching tools. Both approaches are formed by different sub-classifiers—six for template-free and two for template-based, each with a specific view of the protein. Our results show that these ensembles improve the results of each approach individually.

## 1. Introduction

Proteins are present in many biological processes in the cells of living beings, playing different functions, such as transport, growth, and maintenance of the body. They are formed by a sequence of amino acids, which consist of the protein’s primary structure [1]. Amino acids interact physically and chemically with each other, forming three-dimensional structures. The local three-dimensional structure that each amino acid participates in is called the secondary structure, whereas three-dimensional structure that the protein forms is called the tertiary structure [2].

Analyzing the three-dimensional structures of proteins has a great impact on determining the protein functions [3], mainly because each function depends on a specific folding [4], and each protein can exert more than one function [5], as well as the development of new applications, such as drug and enzyme design and biosensors [6,7,8]. Recent work [9] has shown that the prediction of the tertiary structure, directly from the primary structure, can achieve good results, but this task is still open. The most common method in the literature is to first understand the secondary structure and then predict the tertiary structure.

**Figure 1:**
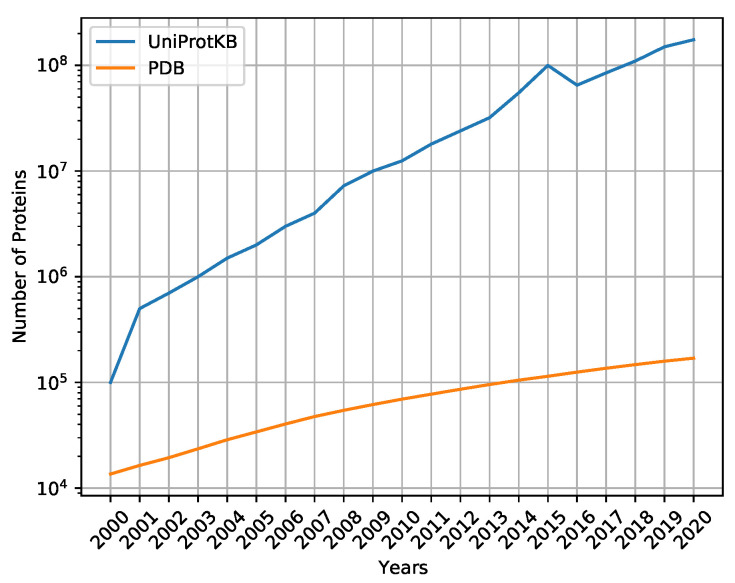
Difference in the number of sequenced proteins deposited in UniProtKB and the number of proteins with determined three-dimensional structures deposited in PDB.

To determine the protein structures, laboratory methods are applied, such as X-ray crystallography, multidimensional nuclear magnetic resonance, and infrared spectroscopy [10], with a complex interpretation of the results. For instance, to determine the secondary structures of the protein, it is necessary to analyze the hydrogen bonding patterns and geometric constraints, and to use the DSSP tool [11]. On the other hand, the number of sequenced proteins grows faster each year, due to advances in gene sequencing [12]. Figure 1 shows the number of proteins sequenced on UniProtKB [13], the main repository of sequenced proteins, and PDB [14], the main repository of proteins with three-dimensional structures defined by laboratory methods, such as secondary and tertiary structures. Thus, computational methods have become important to help predict protein secondary structures efficiently and effectively.

The protein secondary structure prediction task considers that each amino acid can fold into one of eight possible classes. These classes are “B” (residue in isolated beta bridge), “E” (extended strand), “G” (3-helix), “H” (alpha helix), “I” (5-helix), “L” (loop), “S” (bend), and “T” (hydrogen-bonded turn) [3].

The protein secondary structure prediction classifiers in the literature are divided into template-based and template-free classifiers. The template-based classifiers use tools to search and find local alignments between proteins [15], which are usually employed by the Basic Local Alignment Search Tool (BLAST) [16]. The template-free classifiers apply machine learning and deep learning techniques to learn patterns and predict the structures from unseen proteins.

The prediction methods using template-free classifiers for this task started in the 1970s, after Chou and Fasman’s work [17], mainly using statistics and manual rules [18,19]. These first methods for predicting secondary structures of proteins were evaluated in small databases, due to the lack of computational power and the amount of proteins with secondary structures defined by laboratory methods at that time. Furthermore, as they are methods that use simple analyses, most of them were created from a few samples and by manual processes; these methods are less effective than the methods of later decades.

With the development of computer power, the growth of databases, such as PDB [14], CB513 [20], and the biennial Critical Assessment of protein Structure Prediction (CASP) [21], new template-free methods have become popular, such as simple multilayer perceptron neural networks [22,23,24], support vector machines [25], clustering [26], and hierarchical classifiers [27]. At this phase of the problem, of predicting protein secondary structures, the methods employed sliding window techniques to perform the classifications. However, the classifiers did not show a consensus regarding the optimal size of the window used. Furthermore, as these methods can only analyze the local window of interaction between amino acids, longer interactions—that is, amino acids at distant positions in the sequence—are not verified, and can be important to determine the secondary structures.

After 2010, template-free classifiers became the state-of-the-art, mainly using deep learning techniques, such as recurrent neural networks (RNNs) with gated recurrent unit (GRUs) [28] and long short-term memory (LSTM) [29] modules, convolutional neural networks (CNNs), and ensemble techniques. RNN classifiers [30,31,32] receive the entire sequence as input, being able to analyze the chain globally, surpassing the methods that use sliding windows. However, the main negative for this approach involves the computational cost of LSTM and GRU neurons, mainly in bidirectional classifiers, in addition to the problem of vanishing and exploding the gradient.

CNN classifiers [33,34,35] have filters that can analyze several different windows of a sequence. Deep convolutional networks can obtain long-distance information, but local information, gained in the early layers of the network, is lost. With that, several methods have started to use inception blocks, where information from the shallower layer is kept, concatenating the information with the deeper layers. This approach has enabled an advance in the results of the prediction of secondary structures, such as the method developed by Ratul et al. [33], which reached the state-of-the-art in CB6133 [7] and CB513 [20] datasets, with a classifier with inception blocks.

The ensemble techniques for classifiers have shown that they can achieve better results than individual classifiers. Oliveira et al. [12] presented a method for making the ensemble techniques for classifiers, using a bag of bio-inspired optimization algorithms to find weights for each class of each classifier. The authors showed that the genetic algorithm has a greater impact on the final result than the other optimization algorithms. Drori et al. [36] combined several classifiers, using the highest mean prediction value of the classifiers. The approach presented by Drori et al. [36] is penalized by outliers; that is, classifiers that are not ’very sure’ can harm the classification result. Kumar et al. [1] presented a method that uses both RNN and CNN to perform the classification, using CNN as a local classifier and RNN as a global classifier.

Regarding template-based classifiers, BLAST [16] was created in the 1990s, as well as methods using this tool to make predictions of protein secondary structures [12,37,38]. However, template-based methods are not as explored in the literature as template-free classifiers.

Ensemble methods that use evolutionary optimization algorithms have been used in several domains. Haque et al. [39] used a genetic algorithm to find the best combination of algorithms for Alzheimer’s disease classification and face recognition. The method finds the best combination of classifiers, and the fusion is conducted with a simple voting strategy. Prado et al. [40] used a genetic algorithm to find the best combination of classifiers for the energy consumption forecasting problem, while Kausar et al. [41] and Aburomman and Reaz [42] applied the particle swarm optimization algorithm to perform weighted voting of multiple classifiers to predict data from the UCI repository and for detection of TCP/IP connections, respectively. Kardas et al. [43] also employed optimization by evolutionary algorithms for the classification of data from the UCI repository, but unlike the methodology of Kausar et al. [41], the proposed method used genetic algorithms.

The methods used by Prado et al. [40], Kausar et al. [41], Aburomman and Reaz [42], and Kardas et al. [43] found weights for each classifier. These approaches can be disadvantageous if a classifier correctly classifies a class and receives a lower weight in the voting system or a weight equal to 0. Dimilliler et al. [44] compared this methodology with the use of weights equal to 1 or 0 for each class of each classifier during the prediction, making the ensemble using the weighted voting per class and obtaining the highest value. To find the weights, their method applied genetic algorithm. As a result, Dimilliler et al. [44] demonstrated that their approach can achieve better results than finding unique weights for each classifier during the ensemble for a named-entity recognition task of biomedical paper abstracts. Parvin and Alizadeh [45] showed that, by giving weights to each class of each classifier from 0 to 1, the ensemble can reach better results than binary weights, as in the method proposed by Dimilliler et al. [44], with experimental evidence for classification of Farsi digits. Ekbal and Saha [46] also used the genetic algorithm to find weights for each output of each classifier for the weighted voting for the named-entity recognition problem for Indian languages.

In the present study, we investigate six different template-free classifiers and two different template-based classifiers for protein secondary structure prediction. Our template-free classifiers have both local classifications, made with the sliding windows technique (random forest classifier), and global classifiers (RNN classifiers). We also evaluate inception blocks (inception-v4 blocks classifier), inception blocks with GRU recurrent layers (inception recurrent network classifier), as well as classifiers for specific characteristics of the database (a BERT-based classifier and CNN classifier). For the template-based classifiers, we applied a configuration that uses only the best local alignments, but cannot predict all structures for all amino acids, and a general configuration, which can predict for all amino acids.

In addition, we explore the ensemble among template-based classifiers, as well as the ensemble of template-free and template-based ensembles using genetic algorithm optimization. The proposed optimization algorithm finds weights for each class of each classifier. As our main contribution, we explore different representations of the protein features, making our classifiers complementary, obtaining results that surpass the state-of-the-art approaches by 8.2 percentage points on the CB6133 test set and 17.6 percentage points on the CB513 test set.

## 2. Results

In this section, we present and discuss the experimental results of the proposed method on the CB6133 and CB513 datasets.

### 2.1. Template-Free Classifiers

In this subsection, we present the experimental evaluation of the template-free classifiers. We divide this section into results on the CB6133 and CB513 datasets.

#### 2.1.1. CB6133 Dataset

We applied each one of the template-free classifiers to the test set of the CB6133 dataset. The results obtained by each one of them are presented in Table 1. The best individual classifier, RNN, obtained 76.09% of Q8 accuracy on the test set, while the worst classifier was RF, which achieved 64.11% of Q8 accuracy.

Next, we build the ensemble between the template-free classifiers using the genetic algorithm. The ensemble surpassed the best individual classifier, achieving 78.17% of Q8 accuracy, as shown in Table 1. Table 2 presents the weights of each class of each classifier found in the ensemble, showing that BERT received the highest weights in seven of the eight classes, even it not being the individual classifier with the highest Q8 accuracy.

In addition to the quantitative evaluation of the method as a whole, we evaluate the precision and recall of each class, as shown in Table 3. In relation to the precision metric, all classes had more than 0.5 with this evaluation; however, minority classes, such as “B”, “G”, and “S”, had lower values than the majority classes. Regarding the recall metric, again, the minority classes were harmed, due to the large amount of false negatives.

#### 2.1.2. CB513 Dataset

After the experiments on the CB6133, we employed the classifiers on the CB513 dataset. According to Table 4, the best result obtained by an individual classifier was equal to 71.17%, which was achieved by IRN. As we observed with the CB6133 dataset, the worst classifier was RF, which achieved 60.34% of Q8 accuracy.

We then created the ensemble between the template-free classifiers using the genetic algorithm. The result obtained by the ensemble was 73.12% of Q8 accuracy, outperforming the individual classifiers. The weights found by the genetic algorithm showed that the BERT classifier had the greatest impact on the ensemble of template-free classifiers, because this classifier obtained the highest weights in most of the classes, as shown in Table 5.

Next, we analyzed the precision and recall for each class. Table 6 presents these metrics. As we reported on the CB6133 dataset, the precision of minority classes was lower than the majority classes. The recall metric also maintains the same pattern. However, we can highlight the performance of the “B” class, which achieved 0.05 of recall, with many misclassifications.

### 2.2. Template-Based Classifiers

In this subsection, we present the experimental results of the template-based classifiers on the CB6133 and CB513 datasets.

#### 2.2.1. CB6133 Dataset

For the template-based classifiers, we started examining the Q8 accuracy of each classifier on the test set of the CB6133 dataset. The best classifier—that is, the method that obtained the highest Q8 accuracy values among the template-based classifiers, was the general alignments classifier, which achieved 75.96% of Q8 accuracy, as presented in Table 7. From the individual template-based classifiers, we explored the ensemble between them using the genetic algorithm. As a result, the ensemble achieved 78.64% of Q8 accuracy, outperforming the standard template-based classifiers.

Next, we qualitatively analyzed the weights found in the ensemble. According to Table 8, the weights found by the genetic algorithm indicate that, in some classes, specific alignments received the highest weights, such as “E” and “G”, while general alignments received in other classes, such as “B” and “H”.

In addition to method evaluation, we calculate precision and recall for each class. The results obtained are shown in Table 9. Differently from the results of the template-free classifiers, the minority classes, such as “B” and “G”, obtained good precision and recall results.

#### 2.2.2. CB513 Dataset

In the following experiment, we evaluated the general alignments and the specific alignment classifiers on the test set of the CB513 dataset. From the results reported in Table 10, one could observe that the general alignment predictor had better results concerning Q8 accuracy. We also created the ensemble of template-based classifiers. The ensemble obtained better results than the individual methods.

As our ensemble methods found weights for each class of each classifier, we can verify what class received higher weights. According to Table 11, we observed that the specific alignments received the highest values in six of the eight classes when compared to the general alignments. This indicates that this classifier is more important than the general alignments classifier during the creation of the ensemble of template-based classifiers.

With the results of the template-based classifier, we evaluated the precision and recall of each class from the classification in Table 12. As we reported with the CB6133 dataset, the minority classes achieved good results in the precision and recall metrics, differently from the template-free classifiers. We also notice that class “I”, which contained only 30 samples, had precision and recall metrics different from 0.

### 2.3. Ensemble of Template-Free and Template-Based Classifiers

In this subsection, we present and discuss the results of the ensemble of template-free and template-based classifiers on the CB6133 and CB513 datasets.

#### 2.3.1. CB6133 Dataset

After the ensemble of template-free classifiers and template-based classifiers, we made the ensemble between these ensembles. The results obtained by the ensemble of template-free and template-based classifiers surpassed the results achieved by each single configuration, as shown in Table 13.

One important analysis that can be done by our ensemble of template-free and template-based classifiers, involves the value of weights that the fusion method found. According to the weights reported in Table 14, even with less Q8 accuracy than template-based classifiers, template-free obtained the highest weights in six of eight classes.

Next, we analyzed the precision and recall of each class. Table 15 presents these metrics, showing that, in the majority of cases, the ensemble of template-free and template-based ensembles improved the results of precision and recall of each one of the ensembles individually.

**Figure 2:**
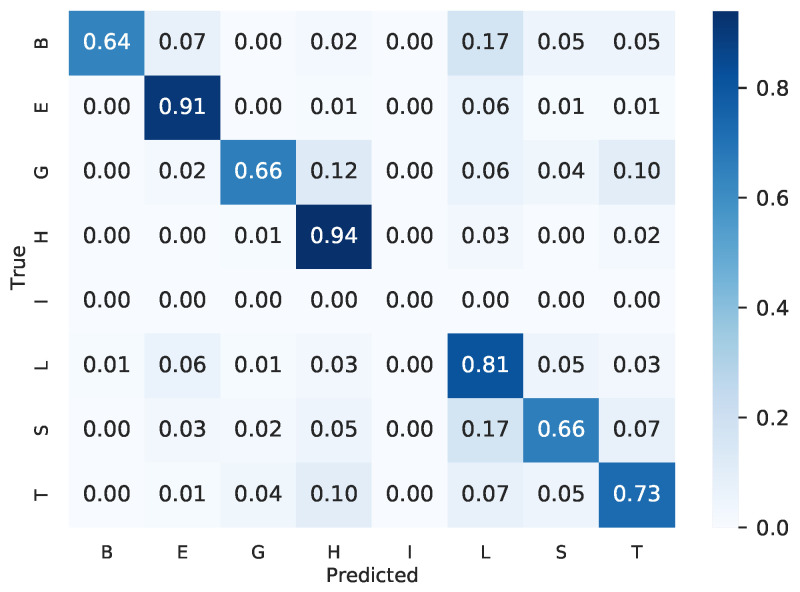
Confusion matrix of the ensemble of template-free and template-based ensembles.

To assess the obtained results, we evaluated the confusion matrix generated by our final ensemble. Figure 2 illustrates the confusion matrix, showing that all classes had at least 64% of accuracy. The secondary structure of minority classes, such as “B” and “G”, obtained less accuracy than the majority classes, such as “E” and “H”, suggesting that if there is more data from these classes, the classifier can achieve better results. There is no structure of class “I" on the test set.

With the result obtained by our ensemble of template-free and template-based classifiers, we can compare the Q8 accuracy obtained by our method with the classifiers in the literature. Our proposed method was able to outperform the state-of-the-art approaches [33] by 8.2 p.p., as reported in Table 16. The basic ensembles (template-free and template-based) also surpassed the state-of-the-art approaches.

#### 2.3.2. CB513 Dataset

Following the experiments, considering the template-free and template-based classifiers on the CB6133 dataset, we conducted the evaluation of the fusion between these classifiers on the CB513 dataset. As in the experiment using the CB6133 database, the ensemble between the two methods reached better results compared to the individual ensembles, as shown in Table 17.

After analyzing the weights of the ensemble, as conducted on the CB6133, the ensemble of the template-free received the highest values in more classes. According to Table 18, only in the “B” and “G” classes did the template-based classifier receive higher weights than the template-free classifier.

We then evaluated the precision and recall metrics for each class of the predictions of the ensemble of template-free and template-base ensembles. The results of these evaluations are presented in Table 19. We conclude that the ensemble, in most of the cases, improved the results compared to the individual ensemble classifiers.

**Figure 3:**
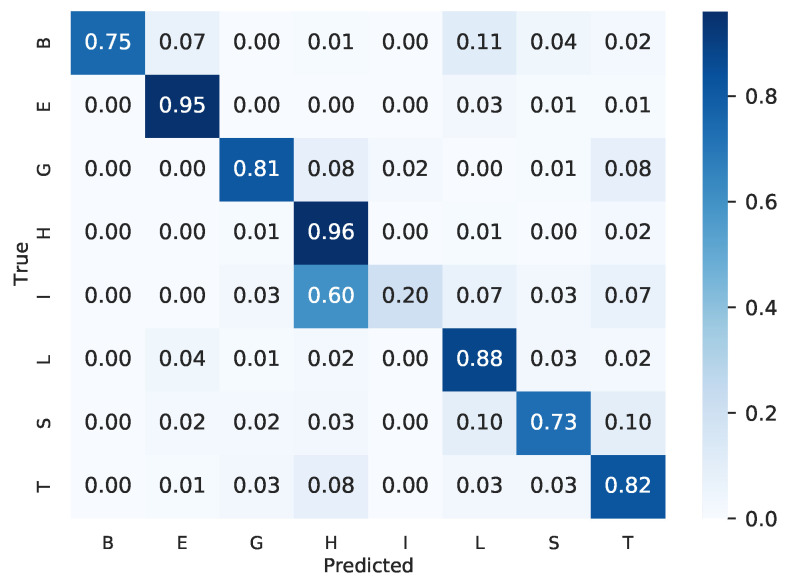
Confusion matrix of the ensemble of template-free and template-based ensembles.

Next, we evaluated the confusion matrix generated by our final ensemble. Figure 3 shows the confusion matrix, which each class, with the exception of class “I”, had at least 73% accuracy. Class “I” has only 30 structures on the test set, and only 6 structures of this class were correctly classified.

Among the methods in the literature, our methods surpassed the state-of-the-art approaches [33]. According to Table 20, our final ensemble surpassed the state-of-the-art approaches by 17.6 percentage points.

### 2.4. Ensemble Evaluation

In this section, we evaluate the results achieved by our ensemble method—genetic algorithm (GA)—compared to other ensemble techniques, using meta-classifiers. We used two different meta-classifiers to create the ensemble, random forest and multilayer perceptron (MLP). Both of them received as input the predictions on the training set of the classifiers that will be combined.

For a fair comparison with our method, we made a grid search for each one of the ensembles, for instance meta-classifier random forest to ensemble BERT. We evaluated different values of the number of trees and the maximum depth for the random forest meta-classifier, and the number of layers, and the number of neurons per layer for MLP. For MLP experiments, we applied the reduced learning rate on plateau and early stopping techniques to boost their performances.

The results obtained by our method and the meta-classifiers are presented in Table 21. Our method (GA) achieved the highest Q8 accuracy on the validation sets on 15 of 18 experiments, showing that this algorithm can obtain robust results and can surpass meta-classifiers on the ensemble tasks.

## 3. Discussion

The objective of this study was to investigate different classifiers with distinct views of the representation of the protein features. Our method has two main ways to make the prediction—template-free and template-based classifiers. Both of them surpassed the state-of-the-art approaches on the CB6133 and CB513 datasets.

For the template-free classifiers, the ensemble between them showed that the most important classifier was BERT, which was the predictor that received the highest weights in more classes. This may have been related to the recent success of this type of algorithm, based on transformers, in different tasks, as in natural language processing (NLP), as shown by BERT, and for image classification, as shown by vision transformer [55].

The results achieved by our template-free classifiers are competitive with the results obtained by other classifiers in the literature. We conjecture that, with more data, our method can obtain better Q8 accuracy because deep learning algorithms have more generalization capability under a variety of data. Further investigation of different types of data augmentation, as augmentation for images, for text, and for tabular data, specific for each one of our classifiers, may help to achieve better results.

The main drawbacks of all of our template-free classifiers involve the lack of explainability of the results; that is, understanding why each classifier makes the prediction of one class and the computational cost of the whole method, which includes training all of the classifiers of one type of classifier (for instance, training ten different bidirectional recurrent networks that compose the RNN classifier), to make the ensemble between this classifier and the template-free classifier. One example of these drawbacks is related to random forest. This classifier is the easiest to verify the decisions made, however, there are 2500 trees to manually verify and understand the choices, making this infeasible.

Our template-based classifier had the highest Q8 accuracy between the basic ensembles (template-free and template-based). We believe that this classifier achieved better results due to the high similarity of the proteins in the test set with the proteins with secondary structures determined by the laboratory methods of PDB. Future works can investigate the impact of limited search databases on the prediction of secondary structures.

The results of the ensemble evaluation section demonstrate that our ensemble method (GA-based) can achieve better results compared to the meta-classifiers. The investigation of other types of ensemble techniques, using deep features, can be a path for future work.

## 4. Materials and Methods

In this section, we describe our template-free and template-based classifiers for predicting protein secondary structures, as well as the ensemble technique employed. We also present the evaluation metrics and the datasets employed in our work.

### 4.1. Template-Free Classifiers

In this subsection, we present our six template-free classifiers—bidirectional recurrent neural network, random forest, inception-v4 blocks, inception recurrent network, BERT, and convolutional neural network.

The execution time required to train each classifier to make predictions on the CB6133 and CB513 datasets, as well as to ensemble the template-free classifiers, is shown in Table 22. In all experiments, we used the Google Colaboratory infrastructure (https://colab.research.google.com, accessed on 22 October 2021).

#### 4.1.1. Bidirectional Recurrent Neural Networks

Recurrent neural networks (RNN) are capable of analyzing and classifying sequences based on past information. However, for the protein secondary structure prediction task, the “past” (the predecessor amino acids) and the “future” (the successor amino acids) impact the secondary structure of an amino acid. Therefore, bidirectional recurrent neural networks are more effective for this task. Even more, this type of classifier can deal with the whole sequence, belonging to the set of global classifiers.

Based on this, we evaluated the use of bidirectional recurrent networks for this task. We analyzed different configurations of RNNs, varying the number of bidirectional layers and the number of neurons per layer. Due to the smaller number of parameters than the LSTM [29], we performed the experiments using GRU [28] memory modules. The best configurations were using 600 neurons per layer and {2, 3, 4, 5, 6} layers.

**Figure 4:**
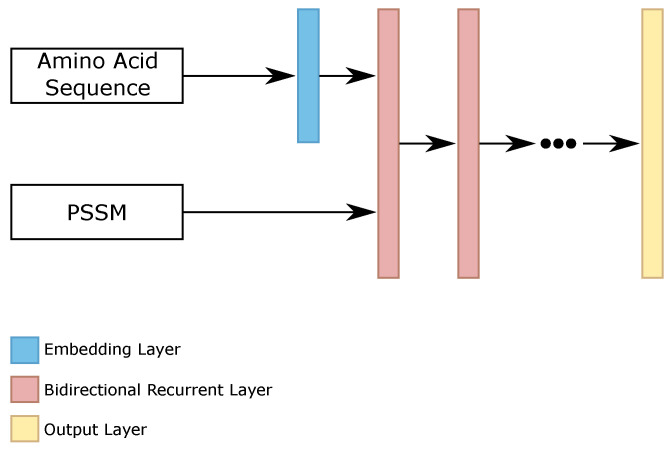
General configuration of each RNN classifier.

As the amino acid sequences are sparse vectors, this is 20 features equal to 0 and 1 feature equal to 1, such as one-hot encoding, we evaluated the presence of an embedding layer for this information. The remaining features, the position-specific scoring matrix (PSSM) information, go directly to the first bidirectional recurrent layer. The results on the validation set showed that this approach helped improve the results. Figure 4 shows the general configuration of our RNN classifier.

For each configuration, we analyzed the ensemble of two networks with the same configuration, with one of them evaluating the sequence in the standard direction, this is in the form that the sequences are present in the files, and another analyzing the inverted way, i.e., from the end to the beginning of the sequence. The probabilities of the two networks are concatenated and normalized, with the sum of all eight classes for each amino acid equal to 1. The final result of the ensemble of each configuration is considered the final prediction of that configuration.

We trained all networks with the TensorFlow [56] framework by 50 epochs, with a learning rate equal to $10^{-4}$, categorical cross-entropy loss function, Adam optimizer, early stopping, and reducing the learning rate on plateau by $10^{-1}$. In the end, we made the ensemble between the five configurations and considered this final prediction as the prediction of the RNN classifier.

#### 4.1.2. Random Forest

The local interaction between close amino acids—different from the global analysis made by RNN—also has an impact on the prediction of protein secondary structures. Between the local interaction classifiers, the methods in the literature usually use sliding window-based predictors; however, each one of them employed different sliding window sizes.

Driven by the local classifiers available in the literature for the protein secondary structure prediction task, we created our local classifier based on random forest. In this classifier, we split the sequence into blocks, and the classifier predicted the secondary structure of the central amino acid. We evaluated different parameters, such as the number of trees, the maximum depth of the trees, and the window sizes, on the validation sets. The best parameters that we found were 500 trees per random forest classifier, with the maximum depth equal to 15, and with 5 different window sizes, from 9, i.e., 4 amino acids before and 4 after the central amino acid, up to 17 (that is, windows of sizes 9, 11, 13, 15, and 17). For padding, we used the same idea as used in the databases—values equal to 0 at the beginning and end of the sequences.

**Figure 5:**
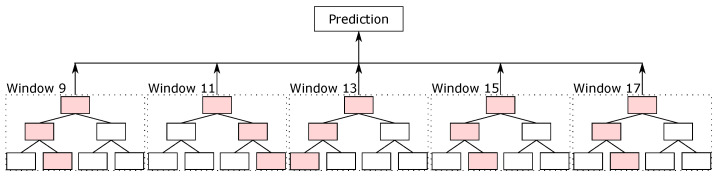
Ensemble of RF classifiers.

With the five random forest classifiers with different sliding window sizes, we made the ensemble between them. The final result made by the ensemble is considered the final prediction of random forest, which we called the RF prediction. Figure 5 illustrates the ensemble of random forest classifiers.

#### 4.1.3. Inception-v4 Blocks

Convolutional neural networks can obtain local information from images from the first layers of the architecture. When the network becomes deeper, the local information gets lost, mainly, due to that, deep layers can understand global and general features of images.

The recent results of inception-based [57,58] protein secondary structure prediction methods [33,49,51] showed that this kind of architecture can aggregate local information, generated by the first layers, and global information, which is generated by deep layers. Driven by this success, we evaluated the newest inception architecture (Inception-v4 [58]) for the protein secondary structure prediction task. As the original inception-v4 was created to make image classification, using 2D convolutions and 2D poolings, we transformed the 2D convolutions and 2D poolings into 1D operations, because our task consisted of analyzing sequences of amino acids, instead of analysis of image parts.

We evaluated the three blocks that composed the inception-v4 architecture, called “block A”, “block B”, and “block C”, stacking from 1 to 10 blocks of the same type. Moreover, we analyzed the use of embedding layers for the sparse amino acid sequence, as we did on the RNN classifier. The best five configurations found on the validation set were using {3, 4, 5, 6, 7} stacked “blocks B”, using the embedding layer for the amino acid sequence.

**Figure 6:**
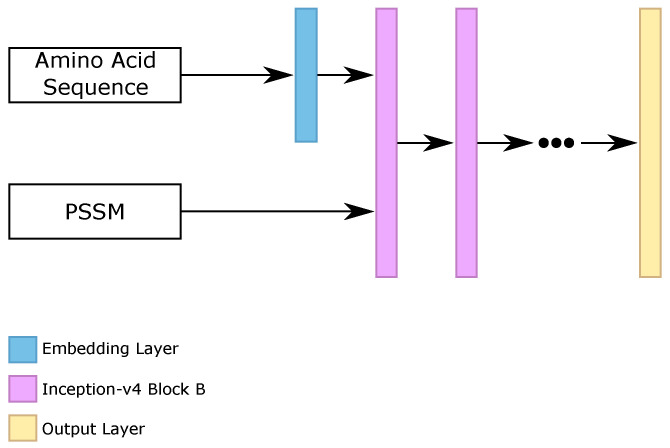
General configuration of each inception-v4 block classifier.

We trained all networks with TensorFlow [56] framework for 50 epochs, with learning rate equal to $10^{-3}$, categorical cross-entropy loss function, Adam [59] optimizer, early stopping, and reducing learning rate by $10^{-1}$ after five epochs, without better results on the validation set. Figure 6 illustrates the general architecture of our inception-v4 block (Iv4B) classifiers.

After the prediction of the five different classifiers (that we called inception-v4 block (Iv4B), we made the ensemble between them. We considered the final ensemble as the prediction of the Iv4B classifier.

#### 4.1.4. Inception Recurrent Networks

Driven by the development of our Iv4B classifier, we evaluated the stacking of bidirectional recurrent layers after the stacked inception-v4 blocks. Our main goal with this method was to use the representations learned by Iv4B classifiers as input to a purely global classifier, in this case, bidirectional recurrent networks.

We employed the five variations of Iv4B, and we evaluated a different number of bidirectional recurrent layers (1, 2, 3, 4, 5), and the number of neurons per layers (100, 200, 300, 400, 500). Our best results on the validation set used 3 bidirectional recurrent layers, with 100 neurons per layer.

**Figure 7:**
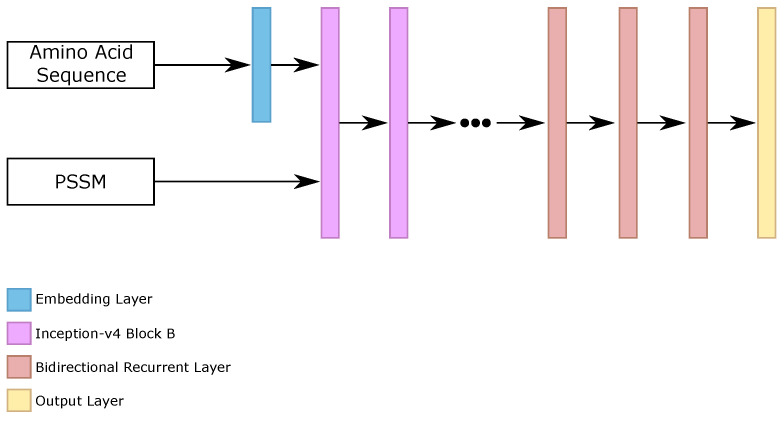
General configuration of each IRN classifier.

We trained all of the networks with TensorFlow [56] framework for 50 epochs, with learning rate equal to $10^{-3}$, categorical cross-entropy loss function, Adam [59] optimizer, early stopping, and reducing learning rate by $10^{-1}$ after five epochs, without better results on the validation set. Figure 7 illustrates the general architecture of this classifier.

Then, we made the prediction between the five configurations of this classifier (that we called inception recurrent networks, IRN). The ensemble is considered the prediction of the IRN classifier.

#### 4.1.5. BERT

Transformer-based [60] architectures achieved good results in many natural language processing (NLP) tasks. The most famous architecture based on transformers is the Bidirectional Encoder Representations from Transformers (BERT) [61]. This architecture surpassed the state-of-the-art approaches in many NLP tasks, such as sentiment analysis, named-entity recognition, sentiment analysis, and text classification. Based on the recent success of BERT, many methods for biological processes began to use BERT for different applications [62,63,64]. Furthermore, as BERT was originally trained on English texts, a new version of BERT became popular for proteins, which was trained on the BFD [65] dataset. This new version, called BERT-prot [64], showed good results on different tasks based on protein classifications.

**Figure 8:**
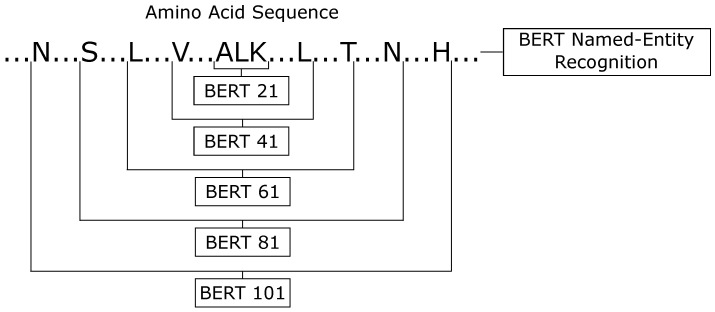
BERT for protein secondary structure prediction task.

Driven by this success of BERT-prot on protein classification problems, we evaluated two different views for the protein secondary structure prediction application. In the first one, we applied the BERT-prot for the named-entity recognition task. In this type of classification, each amino acid is classified in one of each entity, or secondary structure. This first classifier uses all of the sequences as input, so it can analyze the protein as a global classifier. For the second view, we transformed the protein secondary structure into local classification, i.e., we broke the sequences into parts and used each part as a text classification task, classifying the central amino acid. In these two different views, we only used the letters that represented each amino acid and excluded the PSSM information. For text classification, we created padding using special characters. Figure 8 shows all six BERT classifiers.

For the text classification task, we analyzed different window sizes, and the best results on the validation set were obtained by five different configurations (21, 41, 61, 81, 101). All classifiers were fine-tuned by five epochs, using the ktrain [66] package, Adam [59] optimizer, learning rate equal to $10^{-5}$, and early stopping.

In the end, we had six classifiers (one using the named-entity recognition task view, and five using the text classification task view). Then, we made the ensemble between them. The ensemble of BERT-based classifiers was labeled as ’the BERT classifier’.

#### 4.1.6. Convolutional Neural Networks

As our RNN, RF, Iv4B, and IRN classifiers use all of the features from the datasets, i.e., the amino acid sequence and PSSM information, and our BERT classifier only employed the amino acid sequence, we investigated one different classifier that applied only the PSSM features. Considering that this information only has values between 0 and 1, and each amino acid has 21 features for PSSM, the original format available in the datasets is the matrix of Lx21, where *L* represents the size of the sequence. With that, we analyzed the transformation of these characteristics as an image and the usage of convolutional neural networks (CNNs) for the image classification task.

**Figure 9:**
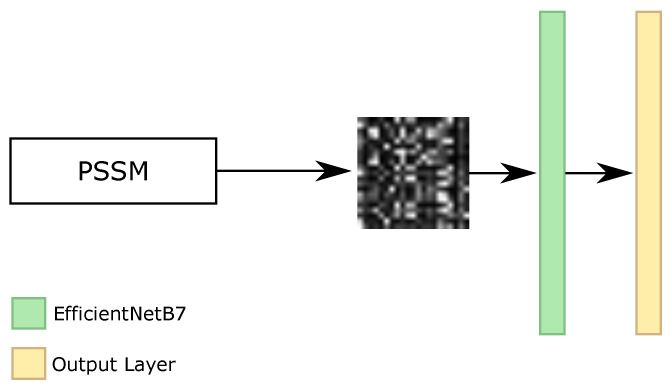
General configuration of each CNN classifier.

With the image representation, we investigated the sliding window classification, i.e., we broke the sequence into parts and we made the classification of the secondary structure for the central amino acid. For the beginning and end of the proteins, we padded with values equal to 0. We evaluated different CNN architectures, such as EfficientNets [67], ResNets [68], and DenseNets [69], as well as the size of the sliding window. The bests results achieved on the validation set were using EfficientNetB7 networks, with images with dimensions equal to 21 × 21, 63 × 63, 105 × 105, and 147 × 147. As the minimum size of input for the networks is 32 × 32, we resized the 21 × 21 image into 63 × 63. Each value of PSSM information was transformed to 3 × 3, 1 × 3, 1 × 5, and 1 × 7 pixels for 21 × 21, 63 × 63, 105 × 105, and 147 × 147 images, respectively. Figure 9 illustrates the general architecture of CNN classifier.

We trained the EfficientNetB7 with TensorFlow [56] framework for 50 epochs, with learning rate equal to $10^{-3}$, categorical cross-entropy loss function, Adam [59] optimizer, early stopping, and reducing learning rate by $10^{-1}$ after five epochs, without better results on the validation set.

Afterward, we made the ensemble between the four networks. The final ensemble between the EfficientNetB7 is considered the CNN classifier.

#### 4.1.7. Ensemble of Template-Free Classifiers

After the predictions of RNN, RF, Iv4B, IRN, BERT, and CNN, we constructed the ensemble between them. We called this ensemble the template-free ensemble.

### 4.2. Template-Based Classifiers

Our template-based classifiers use BLAST to search for homologous proteins. We employed, as a searching database, all proteins with secondary structures determined by laboratory methods from PDB until 2018, and we used the retrieved proteins to make the predictions of the query’s secondary structures. As the protein query could be on the PDB database, we removed it from the retrieved results.

We created two different template-based classifiers. The first method, which we called specific alignments, considers only good alignments between the protein query and the homologies from PDB, while the second method, which we called general alignments, could retrieve general alignments, with fewer restrictions.

The execution required to search for homologous proteins (using BLAST) for specific alignments and general alignments classifiers, as well as for the ensemble of the template-based classifiers, is shown in Table 23. For template-based classifiers, no training step is required, differently from template-free classifiers.

#### 4.2.1. Specific Alignments

For the specific alignments classifier, we evaluated different restriction configurations of the retrieved homologies. We analyzed the number of alignments retrieved, the E-value configuration, and the use of weights to weight the retrieved proteins. We made a grid search with different parameters; the best results achieved on the validation set involved using the first 10 retrieved sequences, with decreasing weights, i.e., the first one received weights equal to 10, the second one received weights equal to 9, and so on, and the restriction of the E-value less than or equal to $10^{-5}$ for the alignments. In the end, the probability of each structure was equal to the alignment voting.

As this classifier only considers good alignments, some amino acids can have no secondary structure predictions. In these cases, we consider that the probabilities of each class are equal to 0.

#### 4.2.2. General Alignments

Our second template-based classifier, general alignments, is more general than the specific alignments classifiers. The general alignments classifier can predict all amino acid secondary structures.

We evaluated different parameters of this classifier, which included E-value configuration, the number of alignments retrieved, the use of local search, i.e., if the amino acid did not receive a prediction, we searched for neighbor amino acids that received a prediction. After the grid search, the best parameters found on the validation set was the E-value less than or equal to 10, using the top 100 retrieval sequences, with decreasing weights, i.e., the first one received weights equal to 100, the second one received weights equal to 99, and so on, and a local search of a size equal to 201, and if the window did not find a prediction, it could increase.

#### 4.2.3. Ensemble of Template-Based Classifiers

After the prediction of the specific alignment and general alignment classifiers, we made the ensemble between them. We called this ensemble the template-based ensemble.

### 4.3. Ensemble of Template-Free and Template-Based Classifiers

After the ensemble of template-free methods and the ensemble of template-based classifiers, we made the ensemble between them, called the final ensemble. This process required 52 min for execution, considering the predictions on the CB6133 and CB513 datasets.

### 4.4. Ensemble Method

We used the method presented in this section to make the ensemble between each classifier, for instance, the ensemble of random forests, between classifiers of the same group, such as template-free classifiers, and the final ensemble between the template-free and template-based classifiers.

Our ensemble method uses the prediction of each classifier that will be fused, and it finds weights for each class of each classifier. For example, if we make the ensemble between five classifiers, the algorithm finds eight weights for each classifier, considering that there are eight possible classes, totaling forty weights. All of the weights were found using the predictions made on the validation set.

The core of our method is the genetic algorithm [70]. The algorithm starts with 2000 individuals (representing the weights that will be associated with each class, for each classifier), with weights generated by uniform distribution, between 0 and 1. In each generation, we select the 100 best individuals by the highest Q8 accuracy to generate 900 new individual throw crossovers. These 1000 individuals (parents and new individuals) generate more 1000 individual throw mutations. At the end of each step, i.e., after crossovers and mutations, we normalize the individual dividing each weight by the maximum weight of this individual. We used 1000 generations and the early stopping technique, which stops the algorithm after 50 generations without better results.

Afterward, the algorithms select the top 100 individuals ordered by Q8 accuracy to make a local search. The top 100 individual generate 100 individual throw mutations. Again, we normalized each individual by dividing each weight by the maximum weight of this individual. We carry out this process by 1000 generations or if the early stopping stops the algorithm. In the end, the best individual, i.e., the best weights, based on Q8 accuracy, are used to make the ensemble between the classifiers.

### 4.5. Evaluation Metrics

We applied the Q8 accuracy, precision, and recall metrics to evaluate our method. Equation (1) shows the Q8 accuracy, which is the most important metric used in the literature to compare different classifiers for the protein secondary structure prediction task.

(1) $$Q8\;\mathrm{Accuracy}=\frac{\sum_{i\in \{B,\;E,\;G,\;H,\;I,\;L,\;S,\;T\}}\mathrm{correct}\;\mathrm{predictions}\;\mathrm{in}\;i}{\sum_{i\in \{B,\;E,\;G,\;H,\;I,\;L,\;S,\;T\}}\mathrm{number}\;\mathrm{of}\;\mathrm{amino}\;\mathrm{acids}\;\mathrm{in}\;i}$$

Precision metric is presented in Equation (2), where TP indicates the number of true positive and FP specifies the number of false positives. This metric evaluates the proportion of positive classifications of positive samples.

(2) $$\mathrm{Precision}=\frac{\mathrm{TP}}{\mathrm{TP}\;+\;\mathrm{FP}}$$

Equation (3) shows the recall metric. In the equation, FN indicates the number of false negative samples. This metric evaluates the proportion of correctly classified positive data samples.

(3) $$\mathrm{Recall}=\frac{\mathrm{TP}}{\mathrm{TP}\;+\;\mathrm{FN}}$$

### 4.6. Datasets

In our study, we used two datasets, CB6133 and CB513. In this section, we present some relevant characteristics of these two datasets.

#### 4.6.1. CB6133 Dataset

The CB6133 dataset is a set of 6133 proteins with sequences up to 700 amino acids, and each protein of this database has a maximum of 30% similarity between them [7].

Each protein of the dataset has a matrix of 700 × 50 features, i.e., the first dimension of the matrix indicates the amino acids (proteins with less than 700 amino acids received padding). For each amino acid that forms the protein, there exist 50 features. The 21 first features indicate the letter that represents the amino acid in the one-hot encoding format (all 20 amino acids, for instance “A” for alanine, plus the special amino acid “X”), i.e., each amino acid only has one value equal to 1 and the remaining are equal to 0. The next 21 features were generated by the position-specific score matrix (PSSM) [16]. The last 8 features are the 8 possible secondary structures in the one-hot encoding format. The padding amino acids have all the features equal to 0.

**Figure 10:**
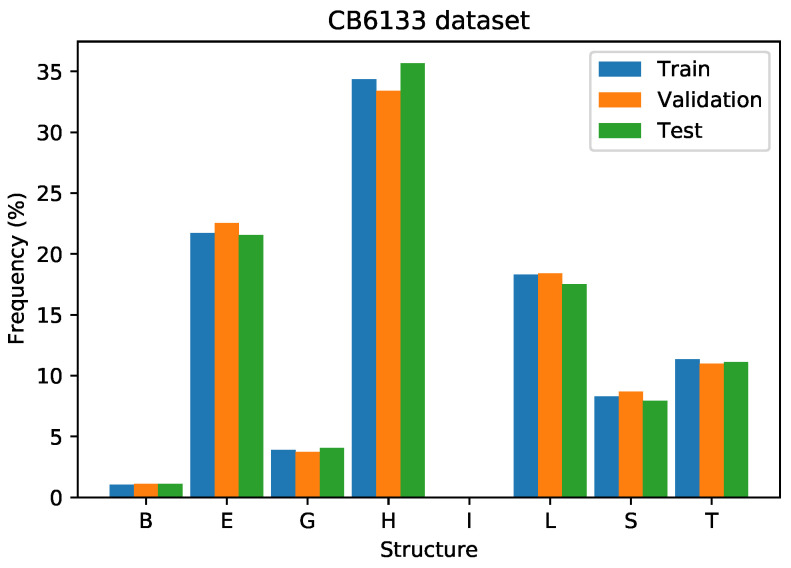
Class distribution on train, validation, and test set on the CB6133 dataset.

We used the same split of the dataset as applied in the literature; that is, 5600 proteins for training, 256 proteins for validation, and 272 proteins for testing. Thus, we are able to compare our results directly and fairly with the results in the literature. Figure 10 presents the distribution of the secondary structures on the training, validation, and test sets, showing that the distributions on these three groups are similar. On the training and validation sets, there are structures of class “I”, while in the test set, there is no data of this class.

For the experiments involving the CB513 dataset, we employed a filtered version of the CB6133 for training and validation. This filtered version of the CB6133 only has proteins with less than 25% of similarity with the proteins of the CB513 dataset. We used the same split of this dataset as applied in the literature, i.e., 5278 proteins for training and 256 proteins for validation.

#### 4.6.2. CB513 Dataset

The CB513 dataset is a set of 513 proteins that were used only in the test [20]. Only one of the 513 proteins has more than 700 amino acids, so we decided to split this unique protein into two proteins, with the first one with 700 amino acids and the second one with the remaining amino acids.

As with the CB6133, all proteins of the CB513 dataset have 50 features, with the first 21 features related to the letter that represents the amino acid in the one-hot encoding format, the next 21 features indicate the PSSM information, and the remaining is related to the secondary structure in the one-hot encoding format. Again, proteins with less than 700 amino acids received padding with all values equal to 0.

**Figure 11:**
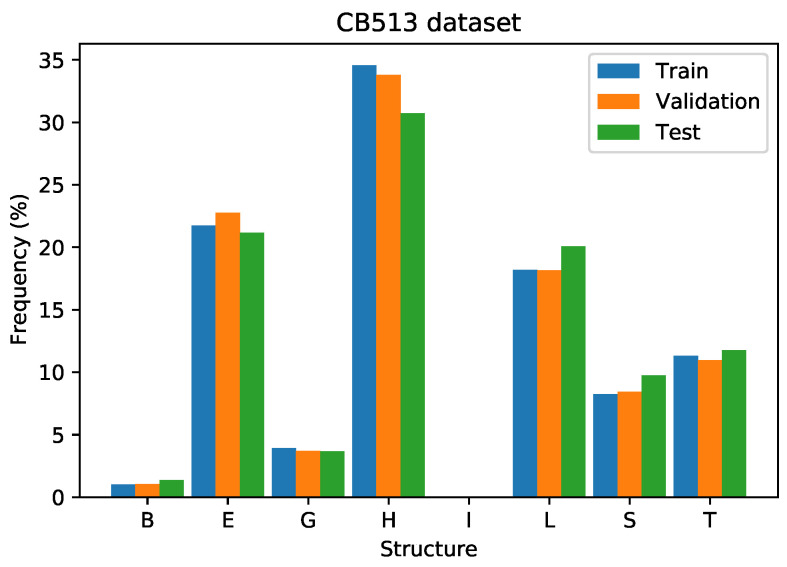
Class distribution on train, validation, and test set on the CB513 dataset.

Figure 11 illustrates the training, validation, and test set distributions of the secondary structures. The training and validation sets employed are from the filtered version of the CB6133 dataset, which has less than 25% of similarity to the proteins on the CB513 dataset. It shows a similar split to the CB6133 dataset. In the CB513 experiments, there exist data of class “I” in the training, validation, and test sets.

## 5. Conclusions

The correct predictions of protein secondary structures significantly impact the determination of protein functions and folding. With advances in sequencing methods and the cost of determining protein secondary structures, the gap between the number of sequenced proteins and proteins with verified secondary structures grows every year. Because of this, several computational methods have been presented in the literature to predict secondary structures, helping to fill this gap.

In this study, we evaluated two different types of classifiers for this task—template-free and template-based. Both classifiers were formed by other classifiers, with a single view of the problem. Moreover, the ensemble of template-free and template-based classifiers demonstrated that they could achieve robust results, outperforming state-of-the-art methods.

## Figures and Tables

**Table 1:** Results of the template-free classifiers on the test set of the CB6133 dataset.

Classifier	Q8 Accuracy (%)
Ensemble of template-free classifiers	78.17
RNN	76.09
BERT	75.80
Iv4B	75.42
IRN	75.29
CNN	72.48
RF	64.11

**Table 2:** Weights found by the genetic algorithm for the ensemble of template-free classifiers on the CB6133 validation set.

Class	RNN	RF	Iv4B	RIR	BERT	CNN
B	0.637	0.303	0.378	0.161	0.314	0.230
E	0.266	0.166	0.105	0.100	0.785	0.104
G	0.399	0.238	0.276	0.243	0.459	0.234
H	0.177	0.291	0.141	0.111	0.538	0.176
I	0.159	0.228	0.179	0.221	0.388	0.276
L	0.275	0.174	0.137	0.066	1.000	0.130
S	0.433	0.244	0.115	0.138	0.940	0.214
T	0.315	0.268	0.145	0.118	0.406	0.181

**Table 3:** Precision and Recall for each class from the prediction of the ensemble of template-free classifiers on the test set of the CB6133 dataset.

Class	Precision	Recall
B	0.68	0.21
E	0.86	0.86
G	0.59	0.44
H	0.90	0.95
I	—	—
L	0.65	0.74
S	0.53	0.44
T	0.69	0.59

**Table 4:** Results of the template-free classifiers on the test set of the CB513 dataset.

Classifier	Q8 Accuracy (%)
Ensemble of template-free classifiers	73.12
IRN	71.17
Iv4B	70.94
BERT	70.24
RNN	69.88
CNN	69.80
RF	60.34

**Table 5:** Weights found by the genetic algorithm for the ensemble of template-free methods on the CB513 validation set.

Class	RNN	RF	Iv4B	RIR	BERT	CNN
B	0.288	0.465	0.554	0.360	0.237	0.310
E	0.156	0.240	0.117	0.202	0.968	0.110
G	0.292	0.429	0.364	0.392	0.589	0.283
H	0.091	0.179	0.253	0.350	0.606	0.131
I	0.320	0.332	0.182	0.228	0.268	0.240
L	0.160	0.147	0.208	0.254	1.000	0.110
S	0.290	0.202	0.248	0.191	0.968	0.164
T	0.245	0.192	0.236	0.398	0.532	0.141

**Table 6:** Precision and recall for each class from the prediction of the ensemble of template-free classifiers on the test set of the CB513 dataset.

Class	Precision	Recall
B	0.55	0.05
E	0.81	0.84
G	0.50	0.40
H	0.88	0.92
I	0.00	0.00
L	0.60	0.71
S	0.58	0.31
T	0.58	0.59

**Table 7:** Results of the template-based classifiers on the test set of the CB6133 dataset.

Classifier	Q8 Accuracy (%)
Ensemble of template-based classifiers	78.64
General alignments	75.96
Specific alignments	69.15

**Table 8:** Weights found by the genetic algorithm for the ensemble of th template-based classifiers on the CB6133 validation set.

Class	Specific Alignments	General Alignments
B	0.791	0.819
E	0.947	0.507
G	1.000	0.473
H	0.859	0.954
I	0.253	0.378
L	0.837	0.613
S	0.961	0.371
T	0.894	0.515

**Table 9:** Precision and recall for each class from the prediction of the ensemble of template-based classifiers on the test set of the CB6133 dataset.

Class	Precision	Recall
B	0.69	0.65
E	0.87	0.84
G	0.71	0.58
H	0.82	0.91
I	—	—
L	0.74	0.70
S	0.65	0.62
T	0.72	0.66

**Table 10:** Results of the template-based classifiers on the test set of the CB513 dataset.

Classifier	Q8 Accuracy (%)
Ensemble of template-based classifiers	89.30
General alignments	86.43
Specific alignments	76.34

**Table 11:** Weights found by the genetic algorithm for the ensemble of template-based classifiers on the CB513 validation set.

Class	Specific Alignments	General Alignments
B	0.738	0.601
E	0.891	0.669
G	0.771	0.488
H	0.597	1.000
I	0.048	0.092
L	0.638	0.611
S	0.560	0.359
T	0.672	0.495

**Table 12:** Precision and recall for each class from the prediction of the ensemble of the template-based classifiers on the test set of the CB513 dataset.

Class	Precision	Recall
B	0.81	0.77
E	0.93	0.96
G	0.78	0.80
H	0.93	0.96
I	0.06	0.47
L	0.74	0.70
S	0.85	0.74
T	0.82	0.81

**Table 13:** Results of the template-free and template-based ensembles on the test set of the CB6133 dataset.

Classifier	Q8 Accuracy (%)
Final ensemble	85.06
Ensemble of template-based classifiers	78.64
Ensemble of template-free classifiers	78.17

**Table 14:** Weights found by the genetic algorithm for the ensemble of template-free and template-based ensembles with the CB6133 validation set.

Class	Ensemble of Template-Free Classifiers	Ensemble of Template-Based Classifiers
B	0.667	0.797
E	0.792	0.423
G	0.834	0.988
H	1.000	0.273
I	0.698	0.465
L	0.772	0.595
S	0.923	0.605
T	0.904	0.616

**Table 15:** Precision and recall for each class from the prediction of the ensemble of template-free and template-based ensembles on the test set of the CB6133 dataset.

Class	Precision	Recall
B	0.74	0.64
E	0.92	0.90
G	0.71	0.66
H	0.93	0.94
I	—	—
L	0.77	0.81
S	0.70	0.66
T	0.77	0.73

**Table 16:** Comparison with the literature on the test set of the CB6133 dataset.

Method	Q8 Accuracy (%)
Final ensemble	85.1
Ensemble of template-based classifiers	78.6
Ensemble of template-free classifiers	78.2
Ratul et al. [33]	76.9
Oliveira et al. [3]	76.4
Drori et al. [36]	76.3
Gou et al. [47]	75.7
Johansen et al. [32]	74.8
Guo et al. [48]	74.2
Zhou et al. [8]	74.0
Zhou and Troyanskaya [7]	72.1

**Table 17:** Results of the template-free and template-based ensembles on the test set of the CB513 dataset.

Classifier	Q8 Accuracy (%)
Final ensemble	89.46
Ensemble of template-based classifiers	89.30
Ensemble of template-free classifiers	73.12

**Table 18:** Weights found by the genetic algorithm for the ensemble of template-based classifiers on the CB513 validation set.

Class	Ensemble of Template-Free Classifiers	Ensemble of Template-Based Classifiers
B	0.811	0.918
E	0.779	0.433
G	0.693	0.828
H	0.984	0.268
I	0.599	0.493
L	0.782	0.513
S	1.000	0.575
T	0.880	0.627

**Table 19:** Precision and recall for each class from the prediction of the ensemble of template-free and template-based ensembles on the test set of the CB513 dataset.

Class	Precision	Recall
B	0.82	0.75
E	0.94	0.95
G	0.77	0.81
H	0.94	0.96
I	0.14	0.20
L	0.88	0.88
S	0.86	0.73
T	0.81	0.82

**Table 20:** Comparison with the literature on the test set of the CB513 dataset.

Method	Q8 Accuracy (%)
Final ensemble	89.5
Ensemble of template-based classifiers	89.3
Ensemble of template-free classifiers	73.1
Ratul et al. [33]	71.9
Busia et al. [49]	71.4
Uddin et al. [50]	70.9
Johansen et al. [32]	70.9
Drori et al. [36]	70.7
Fang et al. [51]	70.6
Zhou et al. [8]	70.3
Gou et al. [47]	70.2
Li and Yu [52]	69.4
Lin et al. [53]	68.4
Wang et al. [54]	68.2
Sønderby and Winther [30]	67.4
Zhou and Troyanskaya [7]	66.4
Hattori et al. [31]	66.0

**Table 21:** Comparison of different ensemble techniques on the validation set of the CB6133 and CB513 datasets.

Classifier	CB6133			CB513		
	GA	RF	MLP	GA	RF	MLP
RNN	77.07	76.04	73.86	75.49	74.70	74.61
RF	64.80	65.03	65.85	65.35	65.57	61.23
Iv4B	75.79	74.85	74.50	76.06	75.12	74.46
IRN	76.00	75.02	74.13	76.35	75.44	75.39
BERT	76.15	75.23	75.23	76.21	75.19	75.36
CNN	73.06	72.09	72.25	72.92	71.59	72.70
Template-free	78.70	77.13	77.03	78.52	77.04	76.57
Template-based	80.18	78.64	80.41	79.80	78.88	79.68
Final ensemble	86.07	79.37	82.29	85.67	80.26	81.27

**Table 22:** Execution time (in minutes) required to train each template-free classifier and to ensemble them.

Classifier	Execution Time (min)
RNN	494
RF	2370
Iv4B	235
IRN	312
BERT	7204
CNN	5285
Ensemble of template-free classifiers	80

**Table 23:** The execution time (in minutes) required to search for homologous proteins for each template-based classifier, and to ensemble them.

Classifier	Execution Time (min)
Specific alignments	58
General alignments	65
Ensemble of template-based classifiers	52

## Data Availability

All classifiers are available at https://github.com/gabrielbianchin/Ensemble-TF-TB-Classifiers (accessed on 22 October 2021).
